# Automated characterization of cell shape changes during amoeboid motility by skeletonization

**DOI:** 10.1186/1752-0509-4-33

**Published:** 2010-03-24

**Authors:** Yuan Xiong, Cathryn Kabacoff, Jonathan Franca-Koh, Peter N Devreotes, Douglas N Robinson, Pablo A Iglesias

**Affiliations:** 1 Department of Electrical and Computer Engineering, Johns Hopkins University, Baltimore, MD 21218 USA; 2 Department of Cell Biology, Johns Hopkins University School of Medicine, Baltimore, MD 21205 USA

## Abstract

**Background:**

The ability of a cell to change shape is crucial for the proper function of many cellular processes, including cell migration. One type of cell migration, referred to as amoeboid motility, involves alternating cycles of morphological expansion and retraction. Traditionally, this process has been characterized by a number of parameters providing global information about shape changes, which are insufficient to distinguish phenotypes based on local pseudopodial activities that typify amoeboid motility.

**Results:**

We developed a method that automatically detects and characterizes pseudopodial behavior of cells. The method uses skeletonization, a technique from morphological image processing to reduce a shape into a series of connected lines. It involves a series of automatic algorithms including image segmentation, boundary smoothing, skeletonization and branch pruning, and takes into account the cell shape changes between successive frames to detect protrusion and retraction activities. In addition, the activities are clustered into different groups, each representing the protruding and retracting history of an individual pseudopod.

**Conclusions:**

We illustrate the algorithms on movies of chemotaxing *Dictyostelium *cells and show that our method makes it possible to capture the spatial and temporal dynamics as well as the stochastic features of the pseudopodial behavior. Thus, the method provides a powerful tool for investigating amoeboid motility.

## Background

The ability of a cell to change shape is crucial for the proper function of many cellular processes, including cell migration. For example, cells of the immune system move in response to pathogen infections by crawling, which involves cycles of protrusions and contractions that deform the entire cell shape [1]. Traditionally, cell motility has been characterized by a number of different parameters [2]. Some, such as velocity, directional persistence and chemotactic index, are determined by the position of the centroid of the cell. Others, including perimeter, area, roundness and body orientation, describe cellular morphology as the cell migrates. These parameters primarily provide global information about motility-induced cell shape changes. Though they can be used to distinguish between strains, they may be insufficient to distinguish phenotypes based on pseudopodial protrusions and retractions, which typify amoeboid motility.

**Figure 1:**
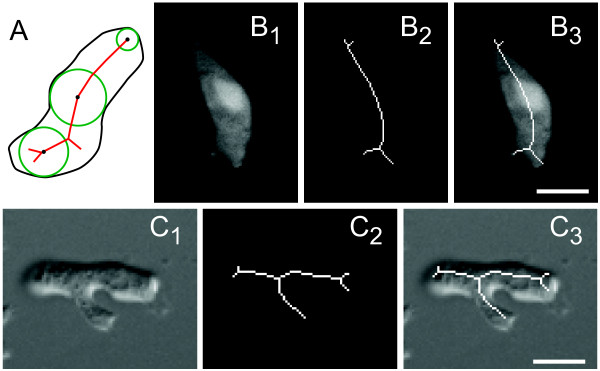
**Skeleton representation of moving cells**. A. The skeleton of a closed region is obtained by finding bitangential circles throughout the cell (three are shown in green). The centers of these circles (black dots) are joined to form the skeleton (red line segments within the region). B. Fluorescent image (B_1_) of a wild-type *Dictyostelium *cell chemotaxing towards the bottom along with the computed skeleton (B_2_) and overlay (B_3_). C. Similar representation for a DIC image. Scale bars represent 5 μm.

Recently, there has been much interest in developing means for processing microscopic images of motile cells and acquiring local morphological information automatically or semi-automatically [2-7]. Here we describe a series of automated methods used to characterize both local morphological changes and statistical features during amoeboid locomotion based on the *skeleton *of a planar shape [8]. Skeletonization, also known as the medial axis transform, is a technique in image processing used to reduce a binary shape into a series of connected lines - the skeleton - that roughly maintains the form of the shape (Figure 1A). This thin-line representation of shape has attracted considerable attention [9,10]. For example, skeletons have been used to measure the lengths and numbers of junctions of tubule complexes in in-vitro angiogenesis assays and to analyze neuronal structures [11]. Part of the technique's attraction is that skeletons of elongated shape patterns, which are frequently observed in many organisms and biological structures, appear to be close to those perceived by humans [12]. Furthermore, the skeleton facilitates shape analysis and uses less data than the original shape. Though skeletonization has long been used to analyze images in cell biology [9,10], it has not been applied to track dynamic information about cellular shape.

We demonstrate that the skeleton can be used to identify pseudopods of moving cells in microscopic images. Moreover, the time-varying evolution of the skeleton can be used to capture the dynamic nature of the pseudopodial protrusions and retractions. We emphasize that the primary goal of this article is not to suggest mechanisms of directed cell motility, but to provide a useful computational tool to analyze amoeboid motility behavior, thus to facilitate the understanding of specific mechanisms in different studies.

## Results

Our method consists of a number of separate steps which we describe here individually.

### Digital image processing

Different imaging techniques are used to generate movies of motile cells: fluorescent, phase-contrast, and differential interference contrast (DIC). Before further image analysis, two steps are performed to process the original cell movies. The first step is *segmentation*, in which cell areas are extracted from their background and cell shapes in each frame are acquired. Based on the image features, different algorithms have been designed specifically for the three microscopy techniques mentioned (Appendix A). The second step is *boundary smoothing*, aimed at removing high-frequency spatial fluctuations of the cell boundary caused by imaging noise. Uniform quadratic B-spline curves, which are smooth curves with continuous first order derivatives and are widely applied in computer aided geometric design, are used to fit cell boundaries [13]. The fitting algorithm is based on the shape-space model and recursive least-square estimations [14] (Appendix B).

### Skeletonization

The skeleton of a shape is defined based on the concept of maximal ball in the context of 2D Euclidean space; straightforward extensions are possible in 3D space [8]. A ball that is inside a closed boundary curve defined by a planar shape is said to be maximal if it cannot be contained in any other larger ball that is also wholly included in the same curve. The skeleton of a planar shape is defined as the medial axis of the shape, which is the locus of centers of maximal balls in that shape. Note that wherever there is a protrusion from the cell membrane, at least one branch occurs in the skeleton (Figure 1A-C).

In practice, because shapes are acquired from digital images, computed skeletons are only approximations of the continuous medial axis. Multiple algorithms that determine the skeleton of a shape defined by a discretized curve have been proposed [15-20]. The medial axis described earlier can be determined based on distance maps, "grassfire" simulations, or Voronoi diagrams. In the distance map, a value is assigned to each point inside the shape corresponding to the distance from this point to the boundary [16,17]. The skeleton points are defined as the ridges in the constructed contour plot. In grassfire simulations, each point on the boundary of the shape serves as the source of a wave that propagates inward with constant speed [18,19]. The skeleton is determined as the singular points generated as the waves collide. These two classes of algorithms are considered to provide equivalent results. Finally, Voronoi diagram-based algorithms use the fact that the skeleton of a shape with discrete boundary points can be approximated by a sub-graph of the Voronoi diagram [20]. The computational load for this class of algorithms is usually greater. Without further processing, skeletons generated by these algorithms are quite sensitive to shape variations since small perturbations in the boundary curve can result in significant changes to the medial axis. To acquire a more meaningful representation of the shape, boundary smoothing (described above) and branch pruning (discussed below) are usually necessary.

Another class of algorithms that create a skeletons which look similar to, but do not necessarily correspond to the medial axis, uses an approach called *thinning*. In these algorithms, the boundary pixels of a region are progressively peeled away without changing the topology of the region, until only a skeletal structure remains [12]. Although this method is preferred in some applications, as it is more robust to boundary variations, cell shape information of interest could be lost. For this reason, we use medial axis skeletons and acquire them using the function *bwmorph *in the Matlab Image Processing Toolbox, the underlying algorithm of which is grassfire simulation (Mathworks, Natick, MA).

### Branch pruning

**Figure 2:**
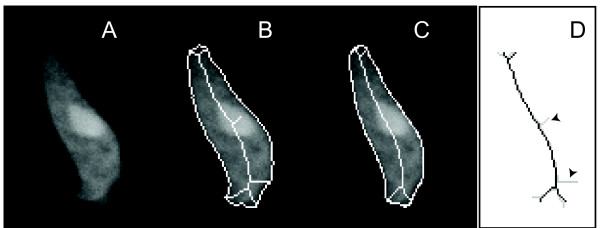
**Boundary smoothing**. A. Fluorescent image of a chemotaxing cell. B. The closed curve shows the edge of the segmented shape before smoothing; the line segments form the skeleton obtained from this shape. C. Smoothed boundary and resultant skeleton. D. Comparison of the original (grey) and post-boundary-smoothing (black) skeletons. Note that several branches deemed to arise from noise fluctuations in the image have disappeared.

**Figure 3:**
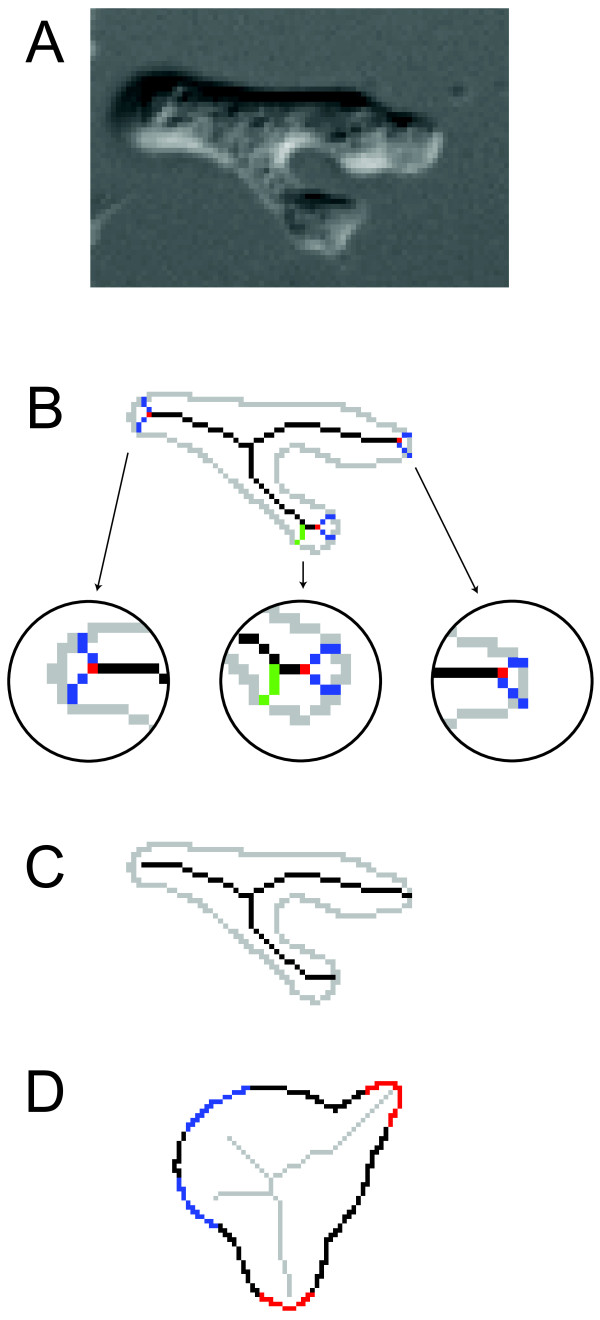
**Branch pruning**. A. DIC image of a moving *Dictyostelium *cell in which the skeleton (B) shows three distinct major branches. Near the boundary, these branches may bifurcate and form smaller branches shorter than the length threshold *p*_threshold _(insets). If the branch is independent; that is, it does not share a root with any other outer branch (middle insert, green branch), we remove it from the skeleton. Otherwise, if the branch shares a root with another branch (blue branches in all three insets), we either combine the two branches (if they are roughly the same length, i.e., the length of the longer branch divided by the shorter one is less than the ratio threshold *r*, three pairs of blue branches in three insets) or remove the shorter branch (when they are of significantly different lengths, i.e., the length ratio is no less than *r*, not shown). C. Skeleton after branch pruning. D. The real protrusion or retraction usually occurs when the branch terminal is close to the boundary (red). If the boundary is locally rounded (blue), the branch will be far away and it is unlikely that a pseudopodial activity happens there.

Boundary smoothing helps to eliminate spurious branches that arise from noisy images (Figure 2). Nevertheless, further branch *pruning *is necessary to map each pseudopod perceived by human eyes to exactly one branch in the skeleton. As the name implies, branch pruning helps to minimize the number of distinct elements in the skeleton description of the cell. Many different pruning methods have been developed [20-24]. All these techniques work by defining a metric of significance to skeleton points. In the case of motility-induced cell shape changes, two criteria are designed to prune spurious branches (Figure 3).

First, short skeleton branches correspond to relatively small variations in shape; conversely, long branches arise from more significant shape features. Thus, the skeleton is pruned by removing small branches. The *outer branches *of a skeleton are defined as the line segments with at least one terminal that is not connected to any other part of the skeleton. The point linking an outer branch with the inner part of the skeleton is denoted as the *root *of this branch (Figure 3B, red). With a preset length threshold of *p*_threshold _pixels and a length ratio *r *> 1 (empirical values given in Table 1), each outer branch shorter than *p*_threshold _is considered:

1. If the branch is independent; that is, it does not share a root with any other outer branch, then it is removed from the skeleton (Figure 3B, green).

2. Otherwise, if the branch shares a root with another branch, then their respective lengths are compared:

▪ If either the length of the longer branch is less than *r *times of the length of the shorter one, or the longer one itself is shorter than *p*_threshold_, then the two branches are combined into a single one arising from the root (Figure 3B, blue).

▪ If the longer branch is longer than *p*_threshold _and longer than *r *times of the shorter one, then the shorter branch is removed and the longer one retained.

If more than two outer branches share a common root, they are compared and if necessary, combined sequentially. To combine two branches, the point at the average position of the two terminals other than the root is identified and linked to the root. By doing this, not only is shortening of the branches by simply removing the two short branches avoided, but it also guarantees that the combined branch represents the approximate center line of a pseudopod (Figure 3C).

**Table 1:** Values of preset parameters in algorithms^1^.

	Variable	Definition	Value used
Branch pruning	*p*_threshold_	Length threshold to identify a short outer branch	1/10 of the length of the cell body^2^
	*r*	Ratio threshold to decide whether to combine two branches or to delete the shorter one	1.5
	*PrDist*_threshold_	Distance threshold to delete a branch far away from the boundary	1/6 of the length of the cell body

Backward tracking	*T*	The maximal length of time apart by which two activities can be considered as from the same pseudopod^3^	50 s
	*α*	Weight on the first order difference in computing the cost function in tracking	0.5
	*β*	Weight on the spatial distance in computing the distance score	0.5
	*R*	Cell radius	5 μm
	*Dist*_threshold_	Threshold on distance score to group two activities into the same pseudopod	1/10 of the perimeter of the cell boundary

^1 ^These values are chosen to minimize the discrepancy between the automated method and manual judgments. For the branch pruning algorithm, representative cells with different sizes and shapes are selected, and their pseudopods during movement are identified by experienced cell biologists. For backward tracking algorithm, manual tracking results from representative movies are independently acquired. Different parameter values are then tested in algorithms, and the best combinations are selected as given.

^2^The "length of the cell body" is computed as the average body length among all the cells from the same experiment. Thus, it is a constant when analyzing movies from the same experiment, but may vary for different experiments.

^3^This parameter needs to be set empirically according to specific frame rate used during imaging; the value here was chosen for movies sampled at 10 s/frame. For all other parameters, the values given here are applied for all our movies, which include different cell types, frame rate, and magnification of imaging.

The second criterion for pruning is based on the local curvature of the boundary curve, which can be determined by the reciprocal of the radius of the osculating circle [25]. Typically, each protrusion or retraction occurs near one point where the local curvature is relatively high compared to other parts of the boundary (Figure 3D, red). Because the terminal point of an outer branch is the center of the circle associated with this boundary point, the distance from the terminal point to the boundary gives a measure of the local curvature. When this distance is big, the boundary has a small curvature locally. Thus, the corresponding branch is unlikely to point to a protruding or retracting region of the cell (Figure 3D, blue). To eliminate these branches, a distance threshold (*PrDist*_threshold_; Table 1) is set and all branches with terminals (other than their roots) farther away from the cell membrane are removed.

### Detecting pseudopodial activity

**Figure 4:**
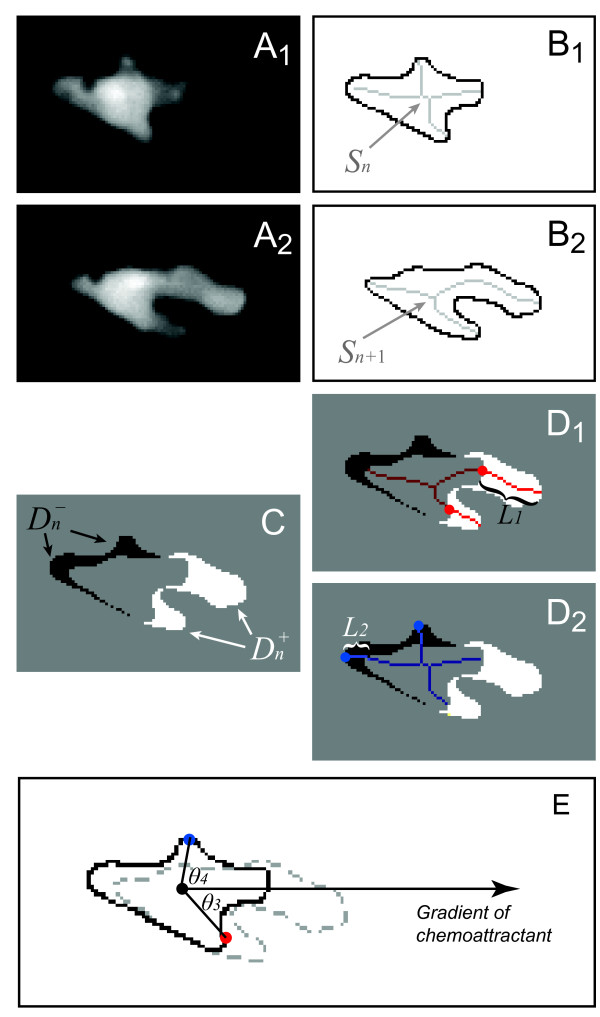
**Dynamic skeletons**. A. Two consecutive images of a chemotaxing *Dictyostelium *cell; A_2 _shows the latter frame. The images are 30 seconds apart. B. Cell boundaries and their respective pruned skeletons: *S*_*n *_for the earlier frame and *S*_*n*+1 _for the latter one. C. By comparing the regions in the two images, a difference map is obtained describing the deformation of the cell from one image to the other. White regions () are growing, while black regions () are withdrawing. D. Branches of the skeleton in the latter frame (red lines) signal protrusion activities if they are pointing at regions in , and branches of the skeleton in the earlier frame (blue lines) signal retractions if they are pointing at regions in . The starting positions of these activities are decided by the points where *S*_*n*+1 _or *S*_*n *_intersects the boundary curve of the earlier frame (red dots for protrusions and blue dots for retractions). The size of an activity is calculated as the length of the part of associated branch that resides in the growing (*L*_1 _in D_1_) or withdrawing (*L*_2 _in D_2_) area. E. The relative angle of a protrusion (*θ*_3_) or retraction (*θ*_4_) is defined when the cell is moving in response to a gradient of chemoattractant, based on the center of the cell in the earlier frame (the black dot) and the starting point of the activity (red dot for protrusion and blue dot for retraction).

As cells move, their shapes deform leading to temporal changes in the corresponding skeletons (Figure 4). We seek to describe this dynamic process and to establish an algorithm to determine whether changes in cell shape correspond to protrusions or retractions. Shape differences refer to the regions where the shape changes during motility from one frame to the next [26]. They can be "positive" or "negative" flow, depending on whether the areas are growing or shrinking. However, owing to image noise and random perturbations of membrane shape, differences are not always well defined. Thus, shape differences are combined with skeletons to detect protrusion and retraction activities.

The process is initiated by segmenting successive images of a moving cell (Figure 4A). Assume that *I*_*n *_and *I*_*n*+1 _are the binary segmentations at the *n*^th ^and (*n*+1)^st ^frames respectively, where the pixels within the cell are set to 1 and those of the background to 0. Denote the pruned skeletons in frames *n *and (*n *+ 1) by *S*_*n *_and *S*_*n*+1 _respectively (Figure 4B). Let *D *be the 2D space where all the image frames of a movie are defined, and *p *be a given pixel in *D*. Define by

and

the expanding and retracting regions of the cell, respectively, from the *n*^th ^to the (*n*+1)^st ^frame (Figure 4C). Note that branches of the skeleton from the (*n*+1)^st ^frame extend out from their root into  and those from the *n*^th ^frame extend out from their root into  (Figure 4D).

A *protrusion *is defined as a region in  that includes a branch of *S*_*n*+1 _(Figure 4D1). The starting position of a protrusion on the membrane in frame *n *is set by the intersection of *S*_*n*+1 _and the boundary of *I*_*n*_, and the length of protrusion between frames *n *and (*n *+1) is given by the length of branch residing in (*L*_1 _in Figure 4D1). Similarly, a *retraction *is defined as a region in  that includes a branch of *S*_*n *_(Figure 4D2). The starting position on the membrane in frame *n *and the contraction length are defined in the same way.

There may be situations where a skeleton branch does not touch the cell boundary. In these cases, we extend the branch to the membrane and compute the extended length as the step size of the activity.

If the cell is moving in response to an external cue, for example during chemotaxis, we are also interested in how the protrusion or retraction is aligned to the chemoattractant gradient. To compute this, the 0° line is defined as the ray starting from centroid of the cell along which the chemoattractant concentration increases the fastest. The angle of an activity is calculated as the angle from the previously defined 0° line to the line linking the cell centroid and the starting point of this activity on the membrane (Figure 4E).

The time of occurrences, the spatial positions in the starting and ending frames, and the angles from the cell center are recorded for all the detected protrusions and retractions. These data are used to describe the dynamics of these activities.

### Backward tracking for pseudopodia lineage classification

It has been argued that different cell strains exhibit dissimilar motility and morphological changes because they extend, retract or split pseudopods in different ways [27,28]. To analyze the changing patterns of pseudopodia, the lineage of all detected protrusions and retractions needs to be determined. To this end, we develop an automated tracking algorithm that clusters activities into distinct groups, each representing the protruding and retracting history of a single pseudopod.

The basic idea of the algorithm is to model the pseudopod dynamics using a second-order autoregressive process [29]. More specifically, the following assumptions are made:

1. The changes in spatial position and angle in activities of the same pseudopod from one frame to the next are much smaller than the distance between two different pseudopods;

2. When either the position or angle of the activity changes, the rate of change tends to be constant.

3. If no other protrusion or retraction is observed in an interval of time after the last recorded activity for an existing pseudopod, a pseudopod has terminated. This takes into account the fact that pseudopods have limited lifetimes.

Because pseudopods are believed to split frequently during movement, they are tracked backwards in time [30]. It is necessary to decide whether each protrusion or retraction detected at a given time point is part of an ongoing pseudopod or of a new pseudopod. To achieve this, a weighted distance metric between activities that takes into account differences in the spatial location (*x*, *y*), the angle (*θ*), and the time (*t*) is defined. More specifically, suppose that two activities *p*_1 _= (*x*_1_, *y*_1_, *θ*_1_, *t*_1_) and *p*_2 _= (*x*_2_, *y*_2_, *θ*_2_, *t*_2_), with *t*_2_>*t*_1 _have been identified and it has been deemed that *p*_2 _is associated with activity *p*_3 _= (*x*_3_, *y*_3_, *θ*_3_, *t*_3_), with *t*_3_>*t*_2_. The distance score between *p*_1 _and *p*_2 _is computed as

The terms Δ_1,2 _((*x*_1_, *y*_1_), (*x*_2_, *y*_2_), (*x*_3_, *y*_3_)) and Δ_1,2_(*θ*_1_, *θ*_2_, *θ*_3_) are the spatial and angular distances, respectively, computed from the equation based on the first- and second-order differences (Appendix C). The values *β *and 1-*β *weigh these two distances, and *R *is the radius of the cell. The term for the time difference inside the square root sign considers that the probability of two activities from the same pseudopod decreases with the time distance between them. Values used for *β *and *R *are given in Table 1. After this distance score is computed from each unassigned activity to all the assigned ones up to *T *seconds away, the scores are sorted and the minimal one selected. If it is under a given threshold, *Dist*_threshold_, the activities are deemed to come from the same pseudopod; otherwise, the unassigned activity is labeled as the latest detected activity of a new pseudopod. Finally, a split is recorded when two activities find the same earlier activity as their common "ancestor" during backward tracking.

Using the methods described above, we calculate a number of quantities for individual pseudopods and single cells (Table 2). Computational results for these parameters and the comparisons between different cell strains are presented in detail below.

**Table 2:** Quantities used to describe morphological behaviors.

Classification	Name	Description
***Individual pseudopods***		
General	Lifetime	The length of time period when the pseudopod has protrusion or retraction activity
	Number of detected activities	The total number of detected activities during the lifetime
	Net speed	The net displacement of local membrane during the lifetime divided by the time length

State dynamics	Protrusion/retraction ratio	Ratio of protrusion/retraction activities out of all detected activities
	Protrusion/retraction speed	Total length of protruded/retracted displacement divided by the total length of protrusion/retraction time
	Protrusion/retraction persistence	The longest running time of consistent protrusions/retractions divided by the lifetime
	State persistence	The longest running time of consistent state (either protrusion or retraction) divided by the lifetime

Angle dynamics	Starting angle	The angle at which the first activity of the pseudopod is detected
	Mean angle	The angle averaged over all activities in the pseudopod
	Linear angle changing rate	The slope of the line that fits best to the activity angle changes
	De-trended angle variance	The variance of activity angles after removal of the linear changing rate

Splitting behavior	Origin	The pseudopod is generated newly or by splitting from existing ones
	Number of splits	How many splits are generated from the pseudopod during its lifetime

***Cell Level***		
Group behavior	Average number of pseudopods	The average number of active pseudopods coexisting on the cell membrane at any given time point
	Ratio of pseudopods from splitting	Number of pseudopods generated from splitting divided by the total number of pseudopods
	Average splitting rate	Total number of splits occurred divided by the time length of the period of movement

Averaged behavior	Average protrusion/retraction persistence	Computed by averaging the corresponding quantities for individual pseudopods over all pseudopods in the cell.
	Average state persistence	
	Average protrusion/retraction speed	
	Average net speed	

### Correlation analysis

Fluorescence microscopy can be used to obtain a quantitative description of the cellular localization of molecular species that are believed to contribute to pseudopod dynamics. The spatial correlation of these data with the locations of pseudopod activities can provide information as to how different proteins contribute to cell motility.

For a given cell during a given period of time, an activity variable, *A*(*k*, *θ*), is defined as the length of the activity if an activity is detected at frame number *k*, representing time, and angle *θ *of the protrusion or retraction activity on the membrane. If no activity is detected, then *A*(*k*, *θ*) = 0. An intensity variable, *I*(*k*, *θ*), is defined corresponding to the relative intensity value at angle *θ *and frame *k *on the membrane. The cross-correlation function between these two signals is given by(1)

where , , ⟨g⟩_*k*, *θ *_represents averaging over time and angles, and ⟨g⟩_*θ *_represents averaging over angles only.

Note that the above definitions are invalid if multiple membrane points correspond to the same *θ*. The situation is usually rare for chemotaxing *Dictyostelium *cells; in our movies no cell exhibited such a shape in any frame. Moreover, even if this situation were present, it would not influence the pseudopod detection and tracking method previously described, since these membrane points are parts of different branches of the skeleton, and each activity is defined not only by the angle from the center, but also by the spatial location (*x*, *y*). Thus, if they represent different pseudopodia activities, they will be identified and analyzed separately.

Assuming that the cell membrane is sampled every Δ*θ *degrees, measuring pseudopodial activity and intensity variables in a movie of *N *frames, the cross-correlation computed from Equation 1 is a matrix of the size (360/Δ*θ*) × (2*N*-1), where each row corresponds to an angle value from - (180-Δ*θ*)/Δ*θ*° to 180/Δ*θ*°, and each column corresponds to a time shifting length from -(*N*-1) to *N*-1 frames. A high absolute value at (Δ*k*, Δ*θ*) means that a linear relationship, either positive or negative, exists between the activities and the intensities located (Δ*k*, Δ*θ*) away. This correlation has also been used to analyze the ordered patterns of spontaneous cell migration [5].

### Illustrating the method

To demonstrate the ability of our methods to characterize cell shape changes during amoeboid motility we implement the steps described above to images of chemotaxing *Dictyostelium *cells. Developed AX3 *Dictyostelium *cells, an axenic laboratory strain, are placed in a shallow gradient of cAMP, and DIC images are acquired at 1 s/frame [Additional file 1]. In this representative cell, 58 pseudopods are detected. Most (60%, *n *= 58) are *short-lived*, lasting no more than 7 s; the remaining *long-lived *pseudopods last at least 9 s and account for over 80% of the total detected activities.

From our analysis, it is clear that the behaviors of individual long-lived pseudopods vary dramatically [Additional file 2]. Pseudopods emerge throughout the membrane; furthermore, the angle drifting of pseudopods is also noticeable [Additional file 3]. While some (22%, *n *= 23) long-lived pseudopods remain near the front or rear of the cell, most (70%) drift away from the front for at least half their lifetime. When a pseudopod shifts from the front to the side or back of the cell, it usually transitions from protrusion to retraction. The size of pseudopods also varies considerably; moreover, the lengths of individual activities from the same pseudopods changes over time [Additional file 1].

**Figure 5:**
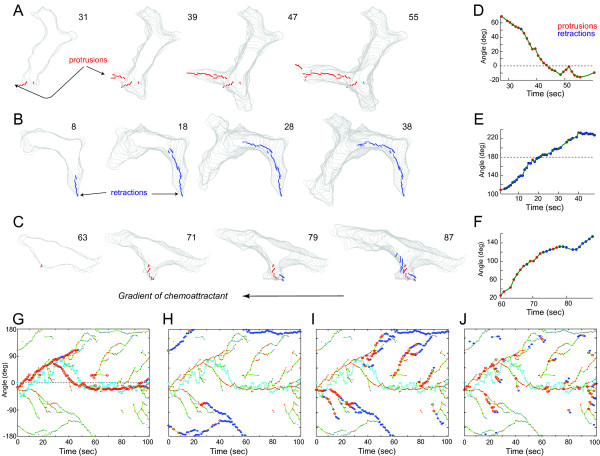
**Individual pseudopods analysis**. A-C. State and angle dynamics for pseudopods with consistent protrusions (A), consistent retractions (B) and protrusions followed by retractions (C). The movie was obtained courtesy of N. Andrew and R. H. Insall. The images show superimposed cell shapes (from the beginning of each pseudopod) and activity trajectories (red for protrusions, blue for retractions). The numbers represent the time (in seconds) from the beginning of the movie. The corresponding activity profiles relative to the chemoattractant gradient are plotted in D-F. In these plots red and blue dots represent protrusions and retractions, respectively, and the green lines join activities coming from the same pseudopod. G-J. The angle dynamics of all pseudopods superimposed by the directions of cell centroid (light-blue dots). Pseudopods with consistent protrusion pattern, consistent retraction pattern, protrusion followed by retraction pattern are highlighted in frame G-I, respectively. All the short-lived pseudopods are highlighted in J.

Despite huge differences in positions and sizes among the long-lived pseudopods, their protrusion/retraction state dynamics fall into three main activity patterns: consistent protrusions, consistent retractions, or alternating periods of protrusions and retractions (Figure 5 and Additional file 4). The average state persistence for this cell is 0.80, which means that pseudopods tends either to protrude or to retract during most of their lifetime. We observed that whether a pseudopod extends or retracts depends on the angle of the pseudopod relative to the chemoattractant gradient. Pseudopods near the front have higher probability of maintaining fast growth (Figures 5D, G). This probability decreases if the pseudopod drifts away from the front (Figures 5F, I). Similarly, the probability of retraction is low at the front of the cell, but increases as the pseudopod approached the back (Figures 5E, H). These observations are consistent with previous results [27,31].

It has been reported that in *Dictyostelium *cells chemotaxing in shallow gradients or moving in the absence of external cues, newly generated protrusions usually split from existing pseudopods [4,27]. Using our method, both the origin of a pseudopod and the number of splits during its lifetime are acquired automatically [Additional file 2]. We find that 74% of all the pseudopods are generated from splitting at an average rate of once every 2.3 seconds. For long-lived pseudopods, 78% are generated from splitting, with an average rate of once every 5.6 seconds.

**Figure 6:**
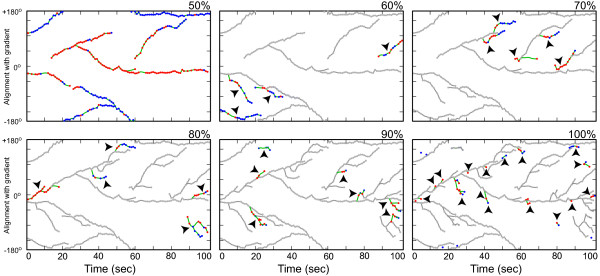
**Hierarchy of pseudopod activities**. Pseudopod activities as a function of time, hierarchically ranked. The panels show all the pseudopods that contribute from 50 to 100% of the cell's total pseudopodial activity. The color scheme of Figure 5D-F is applied to all the pseudopods not included in earlier panels. These pseudopods are also identified with arrow heads.

Previously published results in quantifying pseudopod production rates have shown significant differences. Using manual identification, on average, a new pseudopod is produced every 23 s for AX3 cells moving in shallow gradient [27]. Using automated methods based on local curvatures of sampled boundaries, this number drops to around 14 s for AX3 cells in shallow gradient and 13 s in the absence of chemoattractant [28,32]. Although the experimental conditions might play a role, it is possible that this discrepancy comes from the different ways of identifying pseudopods. To investigate this, the pseudopodia dynamics of the same AX3 cell at different levels are examined (Figure 6). First, the smallest number of pseudopods that cover no less than 50% of all protrusion and retraction activities is selected. Using this criterion, only seven long-lasting pseudopods are identified, of which five contained significant protrusion processes. These pseudopods give a production rate of ~20 s per protruding pseudopod, a number similar to that obtained using manual identification [27]. When the activity percentage level is set to 60%, the number of pseudopods detected with significant protrusions increases to eight. These have an average interval of ~13 s.

### Statistics for phenotype characterization

To show how our methods can be used to distinguish different cell strains, movies from two *Dictyostelium *strains: AX3 and AX3:tsuA mutant are analyzed. AX3:tsuA strain, in which the *Dictyostelium *homolog of the Fused kinase TsuA is knocked out from the parental line AX3, is constructed as previously described [33]. Undeveloped cells from both strains are placed in the gradient of folic acid released from a micropipette containing 1 M folic acid. Images are captured using a 60× objective at 10 s/frame with a spatial resolution of 0.1852 μm/pixel. The pseudopodial behaviors of individual cells are computed and compared between the two strains (Table 3). AX3:tsuA cells show several chemotaxis defects when responding to folic acid. One of the most significant (*p *< 0.05, Student's t-test) defect is the ability to split pseudopods: the ratio of newly generated pseudopods arising from splits decreases 27% and the splitting rate decreases 36%. The other discrepancy is the state persistence. The protrusion persistence decreases 15% and the overall state persistence decreases 10% in AX3:tsuA cells. These data suggest that these cells have difficulty generating consistent protrusions. The net speed of pseudopods, which decreases 27%, can also be related to the lower protrusion persistence: pseudopods tend to wander around instead of pulling out the membrane far away.

**Table 3:** Pseudopod statistics for different cell strains.

Cell strain	(number of cells/frames)	Avg. # of pseudo-pods	% from split	Avg. splitting rate (#/min)	Avg. persistence		Avg. state persistence	Ave. speed (μm/min)		
					**protr**.	**retr**.		**protr**.	**retr**.	net
AX3 (14/1075)	mean	4.2	42	1.54	0.52	0.39	0.82	10.8	9.3	7.8
	STD	0.9	10	0.67	0.06	0.04	0.06	2.2	2.5	2.2
AX3:tsuA (8/756)	mean	3.6	31	0.99	0.44	0.39	0.74	9.3	7.4	5.7
	STD	0.3	10	0.31	0.05	0.04	0.08	1.8	1.3	1.1
*p*-value		0.07	0.02	0.04	0.01	0.94	0.01	0.12	0.05	0.02

AX2 (17/1043)	mean	2.9	34	1.67	0.40	0.36	0.67	16.8	11.3	8.5
	STD	0.7	14	1.09	0.09	0.08	0.13	8.4	4.2	3.1
AX2:dynhp (9/869)	mean	2.8	31	1.66	0.42	0.36	0.72	10.2	8.6	6.6
	STD	0.9	15	1.07	0.07	0.05	0.10	2.2	1.5	1.5
*p*-value		0.67	0.56	0.98	0.54	0.91	0.35	0.03	0.08	0.095

The chemotactic behaviors of developed *Dictyostelium *AX2 cells, an axenic laboratory strain, and AX2:dynhp cells, in which dynacortin, an actin cross-linking protein, is depleted by RNAi, are also compared (Table 3). The mutant strain is constructed as previously described [34]. Cells are placed in the gradient of cAMP released from a micropipette containing 1 μM cAMP. Images are captured using a 40× objective at 5 s/frame with spatial resolution of 0.3077 μm/pixel. The AX2:dynhp cells do not show obvious splitting deficiency. The state persistence is also comparable to that of wild type cells. However, this strain produces pseudopods that protrude and retract more slowly: the protrusion speed decreases 39% (*p *< 0.05, Student's t-test) and the retraction speed decreases 24% (*p *< 0.1, Student's t-test). The net speed of pseudopods decreases accordingly (*p *< 0.1, Student's t-test).

**Figure 7:**
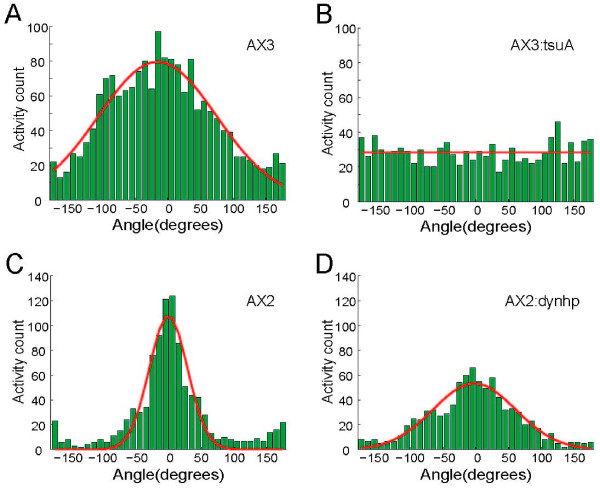
**Protrusion angle distributions for different strains during chemotaxis**. Protrusion angle histogram (green) fitted by a Gaussian curve (red line) for vegetative AX3 cells (A) and AX3:tsuA cells (B), both in the gradient of folic acid released from a needle [33], as well as those for developed AX2 cells (C) and developed AX2:dynhp cells (D), both in the gradient of cAMP released from a needle [34]. The Gaussian fittings yield different values of mean ± STD: -15.5° ± 90.3° for vegetative AX3 cells (A), 0.0° ± 29.1° for developed AX2 cells (C), -2.3° ± 62.8° for developed AX2:dynhp cells (D). The Gaussian fitting for vegetative AX3:tsuA cells does not converge (B). The comparisons of other pseudopod statistics between these strains are summarized in Table 3.

To contrast the efficiency of movement during chemotaxis, it is useful to look at the angle distribution of pseudopods. A histogram of protrusion angles for each of the four strains described above is plotted, and a Gaussian curve to characterize the distribution is fitted (Figure 7). The mean angle of activities indicates whether cells are able to sense the external cues and generate different responses at the front and the back, while the variance measures how efficiently the activities are produced to enable the directed movement of the cell body. The data reveal that vegetative AX3:tsuA cells do not align their protrusions with the chemoattractant gradient. On the other hand, developed AX2:dynhp cells orient their protrusions up the gradient, but not as efficiently as their respective control cells.

### Correlation between pseudopods and molecular localizations

**Figure 8:**
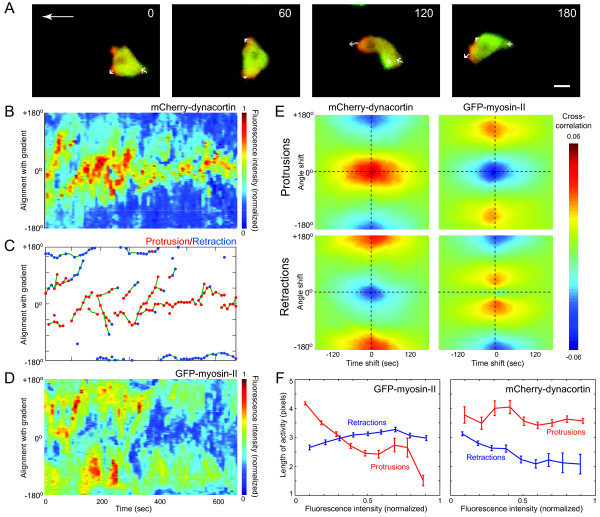
**Localizations of myosin and Dynacortin in chemotaxing cells**. Fluorescent images of a chemotaxing *Dictyostelium *cell expressing mCherry-dynacortin and GFP-myosin-II. A chemoattractant gradient was created using a micropipette needle on the left as previously described [34]. Small arrows placed near cell membrane point to the pseudopodial activities. The numbers at the right upper corners represent the time (in seconds) from the beginning of the movie. The scale bar represents 5 μm. B, D. Fluorescent intensity of mCherry-dynacortin (B) or GFP-myosin-II (D) as a function of time around the cell perimeter for the cell in panel A. The data is normalized between minimum (0) and maximum (1). C. Pseudopod activity as a function of time for the cell in panel A using the same color scheme as in Figure 5D-F. E. Cross-correlations between the two fluorescently-tagged proteins and protrusion or retraction activities as a function of time and angle. Left panels: averaged over 37 cells; right panels: averaged over 100 cells. F. Changes of local protrusion or retraction length in one frame as a function of the local intensity of GFP-myosin-II (100 cells, 4254 frames) or mCherry-dynacortin (37 cells, 1533 frames). Error bars represent standard errors.

To investigate the molecular drivers of pseudopod formation, the correlation between the time and location of pseudopod activities with fluorescently-labeled proteins is calculated. An AX3-based cell strain is created where myosin-II is tagged using GFP, and, simultaneously, dynacortin is tagged using mCherry. At the same time, both endogenous expressions are confirmed to be depleted completely. It is commonly accepted that myosin-II enriched at the posterior during chemotaxis, generating contractile force at the back and squeezing the cell body forward [7,35]. On the other hand, dynacortin is normally concentrated at the leading edge where it is thought to cooperate with actin to influence cortical viscoelasticity [34]. In our study, developed cells are placed in a chemoattractant gradient, and images of chemotaxing cells are obtained using a dual-emission microscope (Figure 8A). The respective fluorescent concentrations are detected around the cellular membrane (Figures 8B, D), and pseudopods are identified and classified as to whether they are expanding or contracting (Figure 8C). The mCherry-dynacortin is found consistently at the front of the cell, aligned with the chemoattractant gradient. In contrast, GFP-myosin-II is found to be mostly at the side.

**Figure 9:**
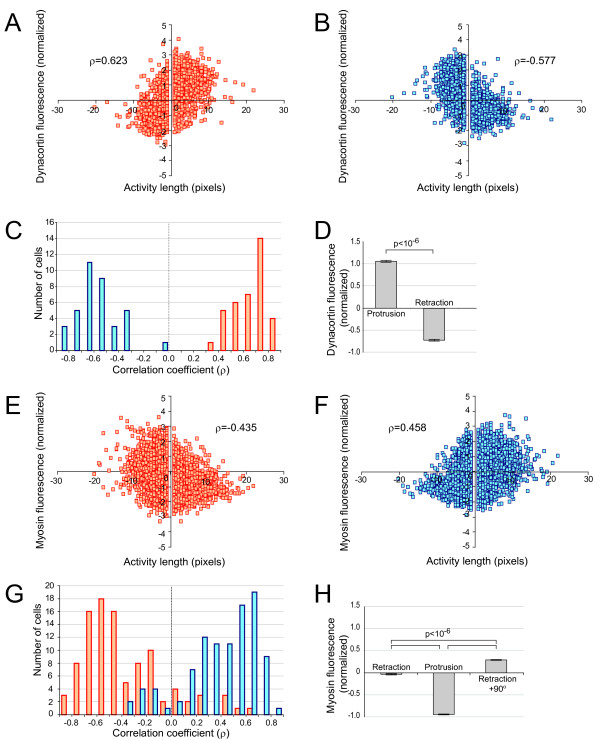
**Correlation analysis between pseudopodial activities and protein localization**. Scatter plot between activity length (positive length implies a protrusion, negative length a retraction, measured in pixels) and dynacortin-mCherry (number of standard deviations above the mean fluorescence around each cell) for all activities both at the site of the activity (A) and 180° away (B). The correlation coefficient (ρ) for each plot is given. These data represent 3333 activities in 37 cells. C. Histogram for the correlation coefficients for each cell at the site of the activity (red) and 180° away (blue). D. Mean dynacortin-mCherry fluorescence at the sites of protrusions and retractions (error bars are SEM). E-H. Similar plots for myosin-II-GFP. These data represent 9019 activities in 100 cells.

To determine how these protein localizations aligned with the pseudopodial activities, the cross-correlation functions (Equation 1) between the two fluorescent intensities and the protrusion or retraction activities are computed (Figure 8E). In nearly all cells displaying good mCherry expressions, peaks in dynacortin concentration occurred at the same time and location as protrusions. On the other hand, retractions and regions of high dynacortin concentration are highly negatively correlated. To probe further these correlations we compute the correlation coefficient between dynacortin fluorescence and activity length to be 0.623 with 99% confidence interval of 0.595 to 0.649 (Figure 9A). On the other hand, when compared to the fluorescence at the other side of the cell (180° away) the correlation is negative: -0.577 with 99% confidence interval of -0.606 to -0.577 (Figure 9B). When measured cell to cell, all cells (*n *= 37) have positive correlations between the length of the activity and fluorescence at the location of the activity, and negative correlation with the activity (180° away) (Figure 9C). The average mCherry intensity at the site of protrusions is significantly higher than at retractions (1.06 vs. -0.72 standard deviations above mean fluorescence intensity at the cell periphery; *p *< 10^-6^, Student's t-test).

A similar analysis of GFP-myosin-II reveals that myosin-II concentrations along the cell membrane are negatively correlated with protrusion activities (Figure 9E-G). However, the relationship between myosin-II and retractions is more complex. In some cases (20%, *n *= 100), myosin-II and retractions are highly correlated. However, in most cases (60%), two peaks of high myosin-II correlation appear almost symmetrically with respect to the 0° line. In the remaining cells, irregular patterns are observed, not showing obvious maxima or minima. The average GFP-myosin-II is significantly higher 90° away from a retraction than at the actual retraction (0.283 vs. -0.034 standard deviations above mean fluorescence intensity at the cell periphery; *p *< 10^-6^, Student's t-test) (Figure 9H). Thus, the relationship between myosin-II concentration and retraction activities may not be simply described as positively or negatively correlated; instead, factors other than myosin-II contraction, such as cortical tension, may contribute to pseudopod retraction [7]. To probe this connection further the length changes for all the detected protrusion or retraction activities are plotted as a function of the local fluorescence intensities (Figure 8F). A range of intensities where the length of the protrusions decreases almost linearly with increasing GFP-myosin-II intensity is observed. On the other hand, the lengths of retractions increased with myosin-II intensity, but with a much smaller slope (Figure 8F). Together, these data suggest that myosin-II's role is not just to create retractions, but to suppress lateral protrusions. Interestingly, although the protrusion speed in AX2:dynhp cells is considerably smaller than that of control cells (Table 3), the localized presence of mCherry-dynacortin did not contribute greatly to the length of the protrusion, but the retraction length did decrease with increasing of dynacortin concentration on the membrane.

## Discussion

Amoeboid motility is a complex cellular process driven by highly-organized cytoskeletal dynamics involving alternating cycles of pseudopodial protrusions and retractions.

In some cases, the subtle differences in motility between different strains may only be revealed by comparison of pseudopod dynamics. However, because of the highly stochastic fluctuations in the behavior of pseudopods, large data sets may be necessary, making manual detection, identification and quantification impractical. In this study we presented an automated method based on skeletonization that detects and characterizes pseudopodial behavior of cells. These algorithms have been illustrated on movies of chemotactic *Dictyostelium *cells.

Several methods for detecting pseudopods automatically or semi-automatically in microscopic cell movies have been proposed previously. In the 3D Dynamic Image Analysis System (3D-DIAS, Soll Technologies), pseudopods are identified based on manual identification of the "nonparticulate" cytoplasm using DIC images [2,7]. In addition to being completely automatic, our proposed method has the advantage that only shape information is needed, thus allowing one to apply this to fluorescent images, making correlation studies like those of Figure 8 possible. Moreover, though all of our examples are illustrated in 2D images, the skeletonization technique can be easily expanded to 3D shapes. Other applications for 3D skeletons have been proposed [36-38].

Pseudopodial activities have also been identified by approximating the local curvature of the boundary using a closed polygon formed from a chain of nodes [4]. This form of detection may depend on the magnification, image quality, as well as sampling density. In contrast, the skeletonization technique uses topological and geometrical information from the whole shape, and takes into account not only the local curvature, but also its relationship to the complete path of the membrane, thus allowing great flexibility in applications.

Related research, based on level set methods, tracks the evolution of virtual markers to determine the spatial and temporal dynamics of a region of interest on the cell membrane [3]. In general, this method requires high resolution imaging in both space and time to deliver topologically consistent solutions. In contrast, our method does not need special boundary markers and can be applied to a relatively broader range of resolutions.

For accurate pseudopod detection, the time resolution of the movies should not be too low, so that a considerable part of the cell body overlaps from frame to frame. The valid range should be decided by the size and speed of the specific cell types of interest. In our case, cells move at ~10 μm/min, and the average length of the cell is 10 μm. The minimal frame rate we use is 10 s/frame, which is equivalent to a distance of ~1/6 of their body length between successive frames. At the same time, the spatial resolution must be selected so that desired pseudopodia activities can be identified at least by eye. The spatial resolution for most of our movies is either ~0.3 μm/pixel when using a 40× objective, or ~0.2 μm/pixel when using a 60× objective. The lower the resolution, the more shape features are lost during imaging, and fewer pseudopodial activities, especially subtle ones, can be captured. This will cause a general problem for all detection method, including manual counting. Other parameters in Table 1 are chosen to minimize the discrepancy between results obtained using the automated method and manual observations of pseudopodial activity. These work well with different cell strains, frame rates and magnifications. However, when tracking significantly different cell movements, other parameters may need to be selected.

One possible drawback of relying on topological information is that skeletonization sometimes cannot detect protrusions with bleb-like structures, since the local curvature at these structures is relatively low compared to typical activities. This problem may also exist in other automated methods based on local curvature calculations. However, in most of our studies, bleb-like structures occur rarely and do not influence the results statistically. For example, there is only one bleb in 101 s in the movie [Additional file 1] at time 59 s, which seems to be a short-lived pseudopod but is missed by the algorithm. Other than the blebs, we do not find shape structures that consistently cause the method to fail in the movies of *Dictyostelium *cells we analyzed. Attempts have been made to characterize the extension of pseudopodia by amoeboid cells in the absence of external cues as well as in shallow gradient of chemoattractant, based on the assumption that spatial differences in chemoattractant receptor occupancy gives rise to biases in the direction of pseudopod extensions [4,28].

However, other studies suggest that the bias might come from pseudopod retractions [27]. Here, we view pseudopodial behavior as a dynamic process that includes both protrusions and retractions, and modeled this dynamic behavior using an autoregressive model - in which the state at a given time depends on the historical activities - for each pseudopod. Our results are consistent with models that suggest that, for a *Dictyostelium *control cell moving in a shallow gradient, a considerable fraction of pseudopods experience both protrusion and retraction, and they tend to retract back when shifting far away from the right direction (Figure 5I). At the same time, the consistent protruding pseudopods may play a more important role in leading the movement of the cell centroid compared to other pseudopods (Figure 5G).

Published results show a discrepancy regarding the production rate of pseudopods, with manual counting reporting less pseudopods than methods that record pseudopods automatically. Our data illustrates that these differences can be attributed to a counting bias. In manual techniques, only the most prominent and persistent protruding activities are identified as pseudopods, whereas automatic methods are able to detect smaller protrusions and retractions. Thus, our method can be applied to quantify both the dominant deformations as well as subtler dynamic perturbations of shape. Our results suggest a higher ratio of "de novo" pseudopods relative to those from splitting during chemotaxis in shallow gradients, compared to previous published results (Additional file 2, [27,32]). We conjecture that this also comes from the fact that not all meaningful boundary activities are captured in these analyses. We note that for cells chemotaxing to a micropipette, the fraction of pseudopods arising from splitting is even lower (Table 3).

By using the cross correlation method to analyze the molecular drivers of protrusions and retractions, we find that dynacortin, as a marker of F-actin, colocalizes with protrusion activities, consistent with the notion that actin polymerization drives protrusions. On the other hand, myosin-II is depleted from the front of the cells, and is enriched at the sides of cells. As myosin-II contributes ~20-30% to cortical viscoelasticity and to cortical tension [39], the localized myosin-II modulated increase in cortical tension at the side of cells may thus help to inhibit lateral pseudopods, as previously suggested [40,41]. This argues that myosin-II has a substantial role in enabling of a polarized morphology seen in cells.

## Conclusions

In this article we propose an automated method to characterize cell shape changes during amoeboid motility. Based on the skeletonization technique, this method makes full use of both the global and local information of cell shape to detect and track pseudopodial protrusions and retractions, and correlates them with molecular localizations. Using the proposed method, the pseudopodial behavior for single cells can be described, the discrepancies among different strains can be disclosed, and the distinct roles of molecules in driving membrane deformation can be revealed. Thus, it provides a powerful tool to investigate amoeboid motility.

## Authors' contributions

YX designed and implemented the algorithms. CK and JFK performed experiments under the guidance of DNR and PND, who provided the experimental materials and platforms, participated in discussions, and contributed to the manuscript. PAI conceived the study, and participated in its implementation and coordination. YX and PAI wrote the manuscript which was read and approved by all the authors.

## Appendix

### A. Image segmentation

We design three different approaches to segment cell areas, according to the imaging techniques used to acquire a specific microscopic movie.

Fluorescence microscopy allows for good contrast between the bright, but of possibly heterogeneous intensity, cell and the dark background. Initial segmentation is achieved by selecting an adaptive threshold intensity that lies between those of the background and cell.

The intensity differences in phase-contrast images come from the anisotropic properties of the medium through which the light travels. Pixel intensities inside the cell are noisy because of the numerous compartments distributed there. Outside the cell, the intensities are considerably smoother. However, a phase ring can be usually observed around the cell periphery, which blurs the boundary and makes the segmentation difficult. To obtain an accurate cell boundary in a phase-contrast image, a deblurring step using the Lucy-Richardson method [42] is first used to remove the phase ring. Next, gradient mappings of the pixel intensity are generated. High gradients are seen in regions where the cell has considerable topological changes and low gradients are seen at the background. Segmentation is achieved by connecting disparate areas using morphological operations [43-45].

In DIC images, because of the existence of a prism-induced shear direction in the image, bright and dark edges coexist on the cell boundary, but little contrast is observed between these edges [46]. Thus, direct methods, either edge detection algorithms or region segmentations, cannot give satisfactory results for cell-shape analysis. A line integration method (line integrated DIC, or LID) has been proposed to transform DIC images to pseudo-fluorescent images [47]. However, this technique can give rise to stripes as a result of the integration of random noise [48]. Deconvolution and/or the Hilbert transform can be used to correct these images, but neither technique avoids introducing distortions in the shape of the cell. Instead, we use a total variation-based approach, in which an optimal image is found by minimizing an energy function defined by the observed image and the properties of the restored image [49]. Two algorithms: one using texture extraction after LID and the other using denoising before LID, are found to be effective and to give similar results [50]. We note that after LID, the pseudo-fluorescent image of the cell will be slightly shifted in the prism-induced shear direction when compared to the cell boundary perceived by human eyes (Figure 1C). However, since this is a constant offset everywhere, it does not affect cell shape or the other variables computed during skeletonization.

### B. Boundary smoothing

The algorithm contains four steps:

1. Segment the image to get the initial boundary;

2. Sample from the initial boundary to get a group of control points for the construction of a B-spline curve, which defines an initial shape estimate and a template in the shape-space;

3. Search for the feature points along the boundary of the initial shape in the normal directions;

4. Use recursive least-square estimation to compute optimal control points according to the positions of the feature points and construct the resultant B-spline curve that fits the boundary best.

As illustrated in Figure 2D, boundary smoothing can eliminate part of spurious branches from the skeleton. We note that splines are appropriate for smoothing as long as the prominent features of a shape are retained. In Figure 2C, the spline faithfully represents the two spots with high local curvatures at the bottom, thus it would not influence the skeletonization results in spite of the missing of small details. However, if a cell has a lot of spiky structures, such as the shape of a fibroblast, and these structures represent the main features of the shape, the smoothing process might be inappropriate as well as unnecessary.

### C. First and second order differences

Let *d*_*t *_be the measured data point (spatial position or angle value) at time *t*. Similarly,  and  are the measurements at time *t-*Δ*t*_2 _and *t+*Δ*t*_1_, respectively. We define two measurements describing how data changes over time. The first

defines the first order difference over two frames. The second

represents the second order difference. These serve as analogues for the first and second time-derivatives, respectively, of the function *d*_*t*_. Thus, the first order difference measures how much the data value changes, and the second order difference measures how much the rate of changes varies over time.

We define a cost function, used in the tracking algorithm that penalizes large changes in both these measures, weighed by constants α and 1-α:

Minimizing the first order difference corresponds to the assumption that changes in spatial position and angle in activities of the same pseudopod from one frame to the next are much smaller than the distance between two different pseudopods. On the other hand, minimizing the second order difference corresponds to the assumption that whenever the position or angle of the activity changes, the rate of change tends to be constant.

Because computing the second order difference for an activity requires three time points, if only two are available, the cost function uses only the first order difference.

### D. Strains used

wt: dynhp Ax2:Rep orf+ (HS1000):: pLD1A15SN:dynacortin RNAi

wt: control Ax2:Rep orf+ (HS1000):: pLD1A15SN

wt: *tsuA*- Ax3 ΔtsuA::pJK1:PHcrac-GFP

wt: control Ax3::pJK1:PHcrac-GFP

*myoII*: GFPmyoII, mCherry dynacortin *mhcA *(HS1):: pBIG:GFP-myosin-II; pDRH:mCherry-dynacortin

## Supplementary Material

Additional file 1 **Movie of a developed *Dictyostelium *cell moving up to a shallow cAMP gradient.** The cell is labeled with detected protrusions (red) and retractions (blue), and pseudopod IDs. The arrows indicate the direction of movement of the cell membrane. The pseudopod IDs correspond to those in Additional files 1 and 2. Click here for file

Additional file 2 **Parameters describing individual pseudopods for a representative AX3 cell chemotaxing in shallow gradient**. Click here for file

Additional file 3 Activity and angle drifting patterns of the long-lived pseudopods in a representative AX3 cell chemotaxing in shallow gradient. Click here for file

Additional file 4 **Movie of a developed *Dictyostelium *cell expressing mCherry-dynacortin and GFP-myosin-II moving up to a micropipette needle releasing cAMP on the left.** The white arrows indicate the detected protrusion and retraction activities. Click here for file
