# Supplementary material for: Automated characterization of cell shape changes during amoeboid motility by skeletonization
Source: BMC Syst Biol. 2010 Mar 24;4:33. doi: 10.1186/1752-0509-4-33 (PMC2864235; doi:10.1186/1752-0509-4-33)
Supplement: Additional file 2 — Parameters describing individual pseudopods for a representative AX3 cell chemotaxing in shallow gradient. [file 1752-0509-4-33-S2.PDF]

Supplementary Table 1. Parameters describing individual pseudopods for a representative AX3 cell chemotaxing in shallow gradient.

ID	Lifetime (s)	Number of		State persistence	Mean angle (degrees)	Angle Shifting Rate (deg/s)	Speed (pixels/s)			Split/ new	# of splits
		protrusions	retractions				Protrusion	Retraction	Net		
1	14	6	4	0.21	53.9	4.2	6.1	-9.1	1.1	split	-
2	4	2	2	0.25	-6.7	3.1	3.5	-1.8	5.3	split	-
3	76	6	67	0.95	-155.5	-1.8	9.1	-11.3	5.8	split	-
4	46	39	1	1.00	-18.3	0.2	9.0	-2.2	7.9	split	6
5	10	6	1	1.00	0.6	2.4	6.6	-1.4	5.2	split	-
6	9	4	2	0.78	-101.7	-2.4	7.4	-5.2	2.6	split	1
7	6	4	2	0.67	-41.9	-5.5	4.0	-4.6	5.7	split	-
8	6	3	0	1.00	-24.7	-2.0	6.4	0.0	5.3	split	1
9	5	3	2	0.60	-74.6	1.5	11.2	-6.4	7.9	split	-
10	1	0	1	1.00	-140.8	0.0	0.0	-7.1	8.5	new	-
11	4	2	0	1.00	97.4	-2.9	2.8	0.0	7.3	split	-
12	9	2	5	0.67	-113.4	-7.6	13.7	-4.6	1.7	new	1
13	2	1	1	0.00	104.1	6.9	4.2	-7.1	1.1	split	-
14	6	1	4	0.83	157.9	0.3	4.1	-10.7	10.8	new	-
15	12	11	1	1.00	12.7	5.1	10.5	-31.1	9.9	split	1
16	2	2	0	1.00	-18.2	-4.7	16.3	0.0	17.0	split	-
17	29	13	14	0.55	106.1	3.9	6.1	-12.2	2.4	split	2
18	2	1	1	0.00	-96.0	-7.8	1.4	-2.8	1.1	new	-
19	12	5	4	0.58	100.5	0.9	6.9	-7.5	2.9	split	-
20	5	3	2	0.60	0.1	7.6	6.1	-3.7	21.7	split	1
21	6	3	1	1.00	75.4	1.3	10.4	-4.2	4.4	split	-
22	13	5	0	0.46	25.7	0.2	3.0	0.0	6.1	new	1
23	3	3	0	1.00	-24.7	-5.6	9.5	0.0	11.8	split	-
24	3	3	0	1.00	-16.9	3.3	10.0	0.0	11.2	split	1
25	1	0	1	1.00	142.5	0.0	0.0	-4.5	5.8	new	-
26	4	2	2	0.25	146.7	-6.3	18.3	-17.2	23.9	split	-
27	34	27	1	1.00	14.7	-2.8	11.0	-2.8	8.6	split	2
28	12	2	8	0.67	158.4	0.3	8.6	-11.5	10.5	split	1
29	13	3	8	0.69	137.4	2.0	7.9	-11.0	7.6	split	1

30	3	0	2	1.00	125.2	4.3	0.0	-6.5	6.7	new	-
31	13	7	5	0.62	97.2	3.0	6.8	-7.2	2.6	split	-
32	48	5	38	0.98	-178.2	2.9	7.8	-13.1	6.2	new	-
33	34	28	4	1.00	75.1	2.4	8.9	-6.6	7.0	split	8
34	9	2	4	0.44	59.8	-2.0	6.8	-3.5	7.7	split	-
35	3	2	1	1.00	-8.1	-20.5	5.9	-3.6	28.5	split	-
36	3	2	0	1.00	92.0	-0.6	4.2	0.0	6.4	split	-
37	16	5	7	0.50	-83.2	-2.5	10.5	-10.3	1.9	split	1
38	2	2	0	1.00	76.6	-5.7	7.6	0.0	17.3	split	-
39	6	3	1	0.67	18.5	-5.9	3.7	-2.8	13.2	split	1
40	3	2	1	1.00	7.1	-6.4	1.4	-4.5	2.2	split	-
41	7	0	4	1.00	153.1	-0.5	0.0	-3.2	6.4	new	-
42	4	3	1	1.00	47.4	-5.7	5.3	-1.4	20.7	split	1
43	5	3	2	0.60	-94.5	-3.2	2.5	-3.9	1.0	split	-
44	5	3	0	1.00	67.5	5.5	7.5	0.0	13.9	split	-
45	1	0	1	1.00	-167.3	0.0	0.0	-10.0	10.5	new	-
46	24	14	3	0.38	-35.3	-1.8	5.8	-9.6	3.4	split	2
47	7	5	1	1.00	-67.6	-6.9	6.5	-5.0	7.6	split	2
48	23	2	15	0.65	-162.0	-0.6	1.9	-6.9	5.6	new	-
49	11	7	0	1.00	19.9	2.2	10.2	0.0	8.0	split	2
50	14	6	6	0.50	-73.8	-7.2	5.3	-6.8	3.7	split	-
51	2	2	0	1.00	44.5	10.6	6.0	0.0	15.7	split	-
52	1	0	1	1.00	-172.6	0.0	0.0	-15.0	16.0	new	-
53	2	2	0	1.00	21.8	12.6	9.5	0.0	17.9	split	-
54	10	9	0	1.00	2.9	4.7	4.9	0.0	5.7	new	2
55	1	0	1	1.00	132.0	0.0	0.0	-3.2	3.4	new	-
56	3	2	0	1.00	-12.2	-0.8	5.9	0.0	10.7	split	-
57	1	0	1	1.00	138.3	0.0	0.0	-6.0	6.5	new	-
58	2	2	0	1.00	-22.0	3.4	7.2	0.0	9.6	split	-
