# Supplementary material for: Automated characterization of cell shape changes during amoeboid motility by skeletonization
Source: BMC Syst Biol. 2010 Mar 24;4:33. doi: 10.1186/1752-0509-4-33 (PMC2864235; doi:10.1186/1752-0509-4-33)
Supplement: Additional file 3 — Activity and angle drifting patterns of the long-lived pseudopods in a representative AX3 cell chemotaxing in shallow gradient. [file 1752-0509-4-33-S3.PDF]

Supplementary Table 2. Activity and angle drifting patterns of the long-lived pseudopods in a representative AX3 cell chemotaxing in shallow gradient.

ID	Activity Pattern			Angle Drifting Pattern		
	Consistent Protrusions	Consistent Retractions	Protrusions → Retractions	Fast: Front → Back	Fast: Back → Front	Slow
1				√		
3		√	√	√		
4	√					√
5	√			√		
6				√		
12			√	√		
15			√	√		
17			√	√		
19			√			√
22	√					√
27	√				√	
28			√			√
29			√	√		
31			√	√		
32		√		√	√	
33	√			√		
34			√		√	
37			√	√		
46			√	√		
48		√				√
49	√			√		
50			√	√		
54	√			√		
